# The Surprising Role of Urea in Promoting CO_2_ Hydrate Formation: Enhanced Molecular Diffusivity via Weakening of the Hydrogen-Bond Network

**DOI:** 10.1021/acs.jpcb.5c04817

**Published:** 2025-09-26

**Authors:** Jun-Wei Hsu, David T. Wu, Shiang-Tai Lin

**Affiliations:** † Department of Chemical Engineering, 33561 National Taiwan University, Taipei 106319, Taiwan; ‡ Institute of Chemistry, Academia Sinica, Nangang, Taipei 115201, Taiwan

## Abstract

Urea is known to act as a kinetic promoter for CO_2_ hydrate formation by reducing the nucleation induction time. Recent molecular simulation studies suggest that urea facilitates CO_2_ hydrate growth by lowering the mass transfer resistance of CO_2_ in water, as evidenced by increased diffusivity of both CO_2_ and water. However, the enhancement of water diffusivity is counterintuitive, given urea’s relatively large size, strong affinity for water, and its tendency to increase the fraction of hydrate-like water structures—conditions typically associated with reduced mobility. To resolve this apparent contradiction, we employ molecular dynamics simulations to examine how urea modifies the hydrogen-bonding network of water, considering both structural and energetic aspects. Our results reveal that urea subtly disrupts the water hydrogen-bond network by competing for bonding sites. The resulting urea–water and adjacent water–water hydrogen bonds are weaker than those in bulk water–water interactions, leading to a locally weakened hydrogen-bond network. This reduction in hydrogen-bond strength lowers the energetic barrier for water diffusion, thereby enhancing molecular mobility. These findings reconcile the seemingly paradoxical increase in both water diffusivity and hydrate-like structuring in the presence of urea, offering new insight into how small organic solutes modulate water structure and dynamics in hydrate-forming environments.

## Graphical abstract

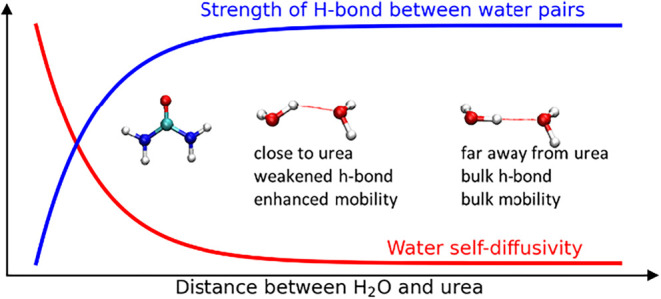

## 1 Introduction

Natural gas hydrates are nonstoichiometric, ice-like crystalline solids formed when water enclathrates small gas molecules, such as methane and CO_2_, under conditions of low temperature and high pressure.^1^ Gas hydrates have attracted considerable attention due to their exceptionally high volumetric gas storage capacity. Notably, the amount of carbon stored in natural gas hydrates is estimated to be roughly twice that of all conventional fossil fuel reserves combined.^2–4^ Recent research has focused on the dual-purpose approach of recovering methane from hydrates while simultaneously sequestering CO_2_,^5–9^ suggesting that gas hydrates may provide a viable pathway toward carbon-neutral energy production.

One of the challenges in many hydrate-related processes is the relatively slow formation of hydrate crystals. The rate of hydrate formation is influenced by several factors, including temperature (degree of subcooling), pressure, concentration of dissolved gas (extent of supersaturation), and the presence of chemical additives.^10–14^ Chemical additives may either alter the thermodynamic driving force (by shifting the equilibrium phase boundary) or change the formation kinetics. For example, urea has been reported to act as both a thermodynamic inhibitor^15,16^ and a kinetic promoter^12,15,17^ for CO_2_ hydrate formation. As a thermodynamic inhibitor, urea reduces the hydrate equilibrium temperature (by 1.86 to 2.49 K in the pressure range of 1.52 to 3.29 MPa at 10 wt % urea concentration.^15,16^ ) by reducing the chemical potential of water in the aqueous phase,^18^ thereby shifting the equilibrium to favor the liquid state. Conversely, both experiments^4^ and simulations^12^ have demonstrated that urea addition enhances hydrate formation kinetics. For example, at 27 bar and 5.3 K subcooling, the addition of 20 wt % urea has been shown to double the nucleation rate.^4^

The dual role of urea as both thermodynamic inhibitor and a kinetic promoter for CO_2_ hydrate formation has motivated many studies to investigate the mechanisms by which urea influences the formation of CO_2_ hydrates. Lim et al.^19^ used mean residence time analysis to observe that urea acts as a molecular mediator and promotes CO_2_ hydrate formation by bridging hydrogen-bond fluctuations and stabilizing the amorphous precursors. In addition to catalyzing interfacial cage formation, Wang et al.^17^ reported that urea enhances mass transport of CO_2_ from the bulk phase to the hydrate interface. This was attributed to a reduction in mass transfer resistance, evidenced by increased self-diffusivity of both CO_2_ and water in urea-containing solutions. Recent study of Sinehbaghizadeh et al.^20^ further supported these findings, noting that urea reduces both heat and mass transport resistances at the hydrate–solution interface and stabilizes partially formed hydrate cages.

Based on these studies, the reduction in mass transport resistance, or equivalently, the enhancement of molecular diffusivity, is considered one of the key reasons for the accelerated CO_2_ hydrate formation in the presence of urea. Dissolved CO_2_ typically reduces the diffusivity of water molecules by promoting the formation of hydrate-like clusters (structured assemblies of water that surround CO_2_) and exhibit slower dynamics. One might therefore expect that urea enhances water diffusivity by disrupting these clusters. Indeed, urea is highly soluble in water and forms favorable interactions with water molecules, which could facilitate such disruption. However, urea is a relatively heavy solute, and water molecules strongly attracted to it would generally be expected to exhibit slower, not faster, dynamics. This presents a paradox: while urea may destabilize CO_2_-induced water structuring, its strong affinity for water and greater molecular mass should slow water mobility. Surprisingly, as we will demonstrate, the fraction of hydrate-like water structures actually increases in the presence of urea. These counterintuitive observations indicate that the enhanced molecular mobility in urea–water–CO_2_ systems cannot be explained solely by the disruption of hydrate-like clusters, and that additional mechanisms must be responsible.

The effect of urea on the hydrogen-bonding network of water has been extensively studied, but with conflicting conclusions. Rupley,^21^ based on viscosity measurements and thermodynamic arguments, suggested that urea disrupts water structure, an interpretation consistent with the positive entropy of dilution. In contrast, diffusion measurements by Mayele and Holz ^22^ showed only a modest decrease in water self-diffusion from 2.30 to 1.78 × 10^–9^ m^2^/s as the urea mole fraction increased from 0 to 0.12 under ambient conditions, indicating minimal structural disruption. Infrared spectroscopy^23^ and ultrafast polarization spectroscopy^24^ studies also support that water retains its dynamic character even at high urea concentrations. In contrast, molecular dynamics (MD) simulations by Idrissi et al.^25^ indicated a reduction in tetrahedrally coordinated water molecules with increasing urea content, implying greater structural disorder. Bandyopadhyay et al.^26^ argued that the local tetrahedral structure remains largely intact if urea is considered a valid hydrogen-bonding neighbor. However, all of these studies were conducted under ambient conditions. The influence of urea on the hydrogen-bonding network of water under hydrate-forming conditions remains largely unexplored.

The objective of this study is to employ molecular dynamics (MD) simulations to explore the effect of urea on the mobility of water and CO_2_ under hydrate-forming conditions. Specifically, we analyze self-diffusivities, hydrogen-bonding networks, and hydrogen-bond energies between water–water and water–urea pairs. In addition, we examine the formation of hydrate-like structures to gain molecular-level insights into the role of urea in promoting hydrate crystallization. Since the structure and energetics of the hydrogen-bonding network are highly sensitive to temperature, our findings should not be directly extrapolated to support or refute conclusions drawn under ambient conditions.

## 2 Simulation Details

Different molecular models, as summarized in Table 1 and Figures S1–S3 in Supporting Information, were created with Materials Studio^27^ for the study of different aspects of the urea-CO_2_-water ternary mixture. Each system was constructed as a homogeneous cubic simulation box with varying concentrations of urea and CO_2_. For the analysis of self-diffusivity and hydrogen bonding, we employed systems containing saturated CO_2_ concentration (*x*_CO_2__ = 0.038) under simulation conditions^28^ (see Figure S6 for CO_2_ solubility), while the urea concentration was set to be comparable to experimental conditions (3.2 to 12.3 wt %).^15,22^ Notably, the simulations were conducted under hydrate-forming conditions (280 K, 45 bar), which differ from typical experimental conditions (298 K, 1 bar). The perfect structure I clathrate hydrate (model C1) was constructed based on Takeuchi et al.^29^

**Table 1:** Molecular Models Used in This Study^a^

model	system	condition	studied properties
A1	1500 H_2_O	270,273,275, 277,280 K and 45 bar	self-diffusivity, hydrogen bonds, hydrate-like structure
A2	1500 H_2_O + 60 CO_2_		
A3	1500 H_2_O + 63 urea (*w*_urea_ = 12.3 wt %)		
A4	1500 H_2_O + 63 urea + 60 CO_2_ (*w*_urea_ = 12.3 wt %)		
B1	1000 H_2_O + 10 urea (*w*_urea_ = 3.2 wt %)	280 K and 45 bar	distance-dependent water–additive interactions
B2	1000 H_2_O + 10 CO_2_		
C1	368 H_2_O + 64 CO_2_		perfect hydrate

a Urea weight percent is defined as *w*_urea_ = *m*_urea_/(*m*_urea_ + *m*_H_2_O_) × 100%.

The initial structures typically undergo energy minimization using the steepest descent algorithm, followed by a short 200 ps NVT simulation at 200 K to relax residual stresses. The system is then gradually heated at a rate of 0.5 K/ps to the desired temperature while pressure coupling is applied, followed by a 1–10 ns NPT equilibration step before proceeding to longer production runs (11–50 ns) at the target conditions. The molecular dynamics simulation package GROMACS^30^ is used for all MD simulations. The leapfrog algorithm^31^ was used to integrate Newton’s equations of motion, with an integration time step of 1 fs. Lennard–Jones potential and Coulomb energies were calculated with a cutoff of 9.5 Å. The Particle-Mesh Ewald method^32^ and dispersion corrections were applied for long-range interactions.^33^ The system temperature was controlled using the Nosé-Hoover algorithm^34^ with τ_t_ = 1 ps, and the pressure was controlled using the Parrinello–Rahman algorithm^35^ with τ_p_ = 10 ps. The TIP4P/Ice potential^36^ was used for water, the EPM2 potential^37^ for CO_2_, and OPLS-AA potential^38,39^ for urea. This choice of force field parameters has been shown to provide many hydrate properties that are in good agreement with experiment.^38–40^ The force field parameters and their validation are summarized in the Supporting Information.

## 3 Results and Discussion

The main focus of this work is to investigate the mechanism by which urea promotes molecular diffusion of CO_2_ and water under hydrate forming conditions. The enhanced molecular diffusion was found to be a key factor for its role as kinetic promoter for CO_2_ hydrate growth.^17^ The enhanced molecular diffusion was first confirmed with the mean-square displacement (MSD) analysis,^41^ and the role of urea is further analyzed through the hydrogen-bond structure and strength. Simulations were performed at 45 bar and 280 K, which is 5 K below the freezing temperature of CO_2_ hydrate based on the force field (Table S1, SI). Each simulation case was repeated at least 10 times to evaluate the data uncertainty.

### 3.1 Urea Accelerates Local Water Diffusion

The self-diffusivity of water and CO_2_ in the ternary mixture of water-CO_2_-urea (Models A1–A4) at 45 bar and 280 K are summarized in Table 2. As can be seen, the presence of CO_2_ decreases the diffusivity of H_2_O (from 6.40 × 10^–6^ to 4.26 × 10^–6^ cm^2^/s). This is expected as water forms hydrate-like cage structures in the presence of CO_2_, which slows the average mobility of water molecules. The addition of urea increases the diffusivity of H_2_O (from 6.40 × 10^–6^ to 6.92 × 10^–6^ cm^2^/s without CO_2_, and from 4.26 × 10^–6^ to 4.97 × 10^–6^ cm^2^/s when CO_2_ is present) and also that of CO_2_ (from 4.46 × 10^–6^ to 4.83 × 10^–6^ cm^2^/s). This effect is counterintuitive, as one would typically expect water diffusivity to decrease due to the presence of heavier molecules with strong affinity for water.

**Table 2:**
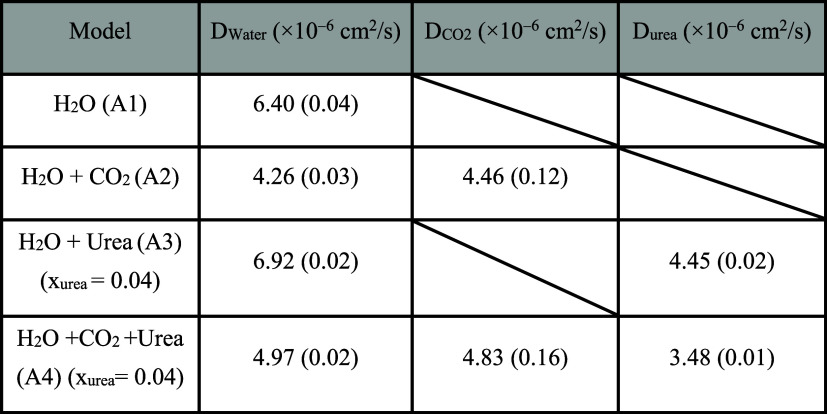
Self-Diffusivity of Different Molecules at 45 bar and 280 K^a^

a Numbers in parentheses represent the standard error (SE) estimated from 12 independent runs; the urea mole fraction is defined as *x*_urea_ = *n*_urea_/(*n*_urea_+ *n*_H_2_O_).

To further investigate the spatial dependence of water diffusivity near additives, we decomposed the velocity autocorrelation function (VACF)^42^ according to the minimum distance between each water molecule and its nearest additive molecule. Specifically, the radial direction from the additive is discretized into equal sized bins (with width Δ*r*), and an indicator function χ_k_(r_i_) to extract the contribution of water molecule *i* to the VAC

1 $$\chi_{k}\left(d_{i}\left(t\right)\right)=\{\begin{array}{l} 1,,r_{k}<d_{i}\left(t\right)\leq r_{k+1}\\ 0,,otherwise \end{array}$$

where *r*_*k*_ = *k*Δ*r* with *k* = 0, 1, 2, etc. *d*_*i*_(*t*) is the distance of water molecule *i* to the nearest additive molecule. The corresponding local diffusivity is then defined

2 $$D_{k}=\frac{1}{3}\int_{0}^{\infty }\left\langle \frac{\sum_{i=1}^{N}\chi_{k}\left(d_{i}\left(t\right)\right)\left(v_{i}\left(t\right)\cdot v_{i}\left(t+\tau \right)\right)}{\sum_{i=1}^{N}\chi_{k}\left(d_{i}\left(t\right)\right)}\right\rangle d\tau$$

where *N* is the total number of water molecules in the system. This approach allows us to quantify water diffusivity as a function of distance from the additive either CO_2_ or urea.

Simulations were conducted at 280 K and 45 bar for 11 ns, with the final 1 ns used to analyze the VACF. Each case was repeated 18 times using Models A1, B1, and B2. To ensure a consistent basis for comparison, we used a reduced additive concentration (approximately one-fourth of that in models A2 and A3), which lowers the likelihood that a single H_2_O molecule is surrounded by multiple additive molecules within its first solvation shell (∼0.4 nm). This reduces local concentration fluctuations and potential bias in spatially decomposed VACF analysis. This setting is particularly important for CO_2_, as high concentrations tend to promote phase separation and bubble formation. As shown in Figure 1, water self-diffusivity increases as it approaches urea, and approaches bulk value (red horizontal line) when the separation distance is greater than 1.0 nm. The enhancement of water diffusivity can be about 16% higher than the bulk value when water approaches urea to within 0.4 nm, where the water molecule is within the first solvation shell of urea (see the radial distribution between urea and water molecules is analyzed in Figures S12–16 of the Supporting Information). In contrast, self-diffusivity of water molecules near CO_2_ is lowered, and can becomes 74% of the bulk value when water is within 0.4 nm of CO_2_.

**Figure 1:**
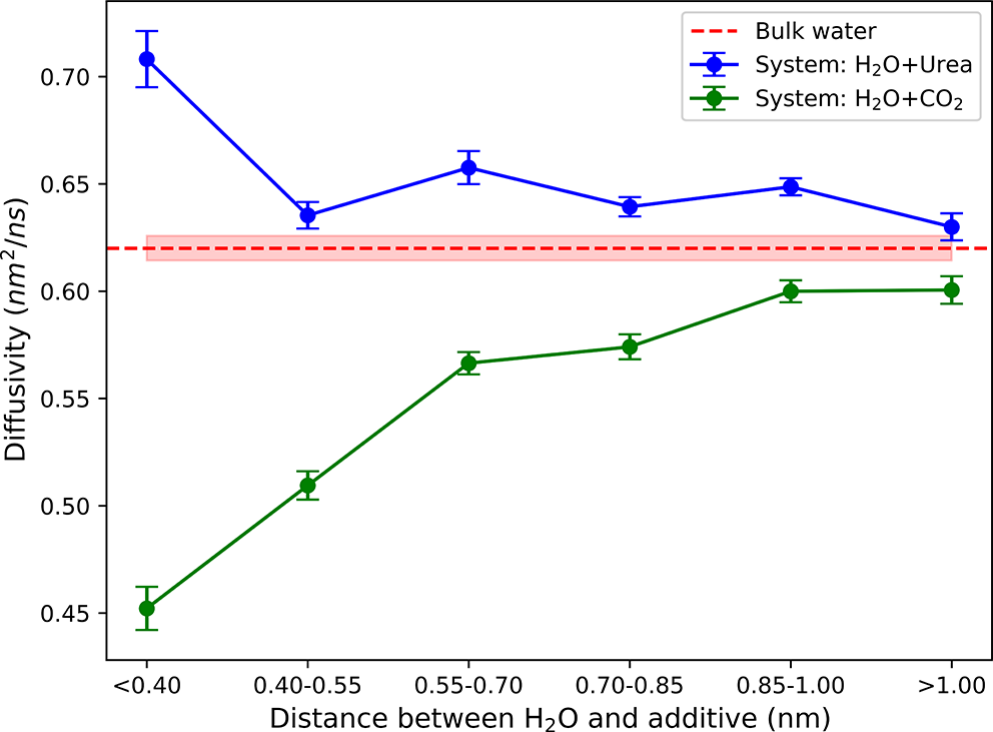
Water self-diffusivity as a function of separation distance from additives: urea (blue circles) or CO_2_ (green circles). The uncertainties are calculated from 18 independent runs.

### 3.2 Urea Reduces the Energy Barrier of Diffusion

To investigate the origin of the enhanced molecular diffusivity due to urea, we first determine the diffusion energy barriers from simulations at 45 bar and 5 temperatures between 270 and 280 K using Models A1–A4. The diffusivity data are calculated from 50 ns NPT simulations after the systems have reached equilibrium. Twelve independent runs were conducted to collect the statistical data. Since the temperature range is small, we assume that the temperature dependence of diffusivity follows the Arrhenius equation, as shown in eq 3.

3 $$D=Ae^{-E_{a}/\mathrm{RT}}$$

where *E*_a_ is the energy barrier for diffusion. Figures 2 and 3 illustrate the natural logarithm of diffusivity (ln *D*) plotted against the reciprocal of temperature (1/*T*) for H_2_O and CO_2_, respectively, in different solutions. The slopes of linear regression to these data give the diffusion energy barrier of water and CO_2_ in different solutions. The results, summarized in Table 3, indicate that the presence of CO_2_ increases the diffusion energy barrier of H_2_O by approximately 19% (*E*_a_/*R* increased from 3425 to 4071 K), likely due to the formation of hydrate-like structures. In contrast, the presence of urea reduces the diffusion barrier for H_2_O by about 20% (*E*_a_/*R* reduced from 3425 to 2748 K). On the other hand, the presence of urea reduces the diffusion energy barrier for CO_2_ by approximately 37% (*E*_a_/*R* reduced from 4098 to 2982 K). It is worth noting that the higher uncertainty in CO_2_ diffusivity observed in Figure 3, compared to that of water (Figure 2), arises from the fewer number of CO_2_ molecules at low concentrations.

**Figure 2:**
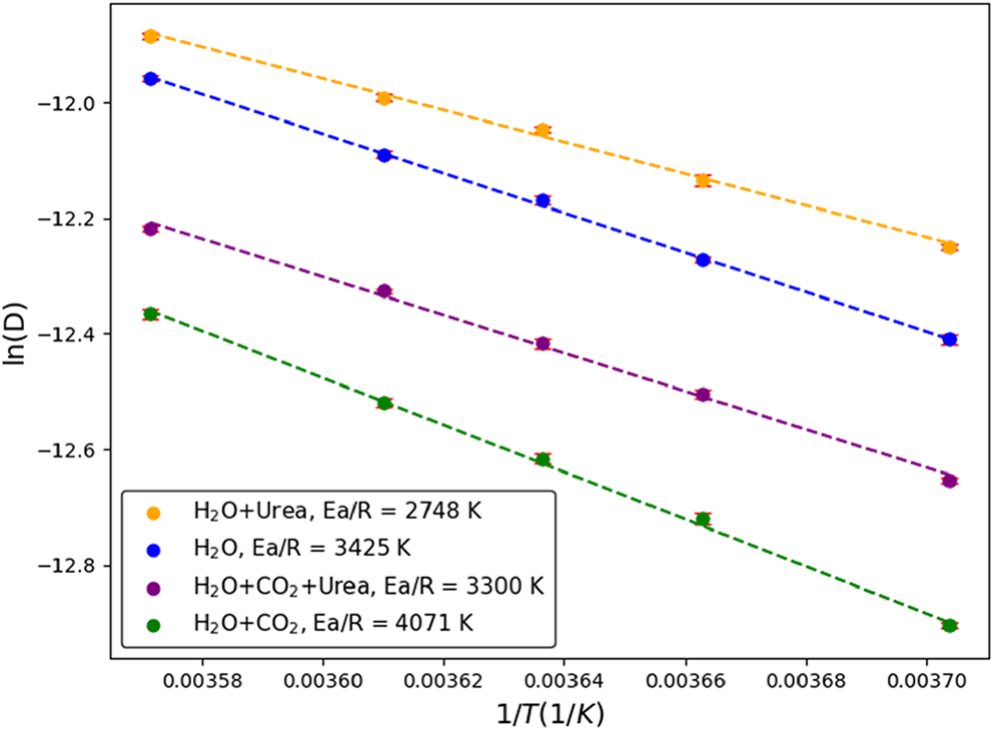
Variation of the natural logarithm of the self-diffusivity of water as a function of the reciprocal temperature in different systems: pure water (blue circles), water with dissolved urea (yellow circles), water with dissolved CO_2_ (green circles), and water with dissolved CO_2_ and urea (purple circles).

**Figure 3:**
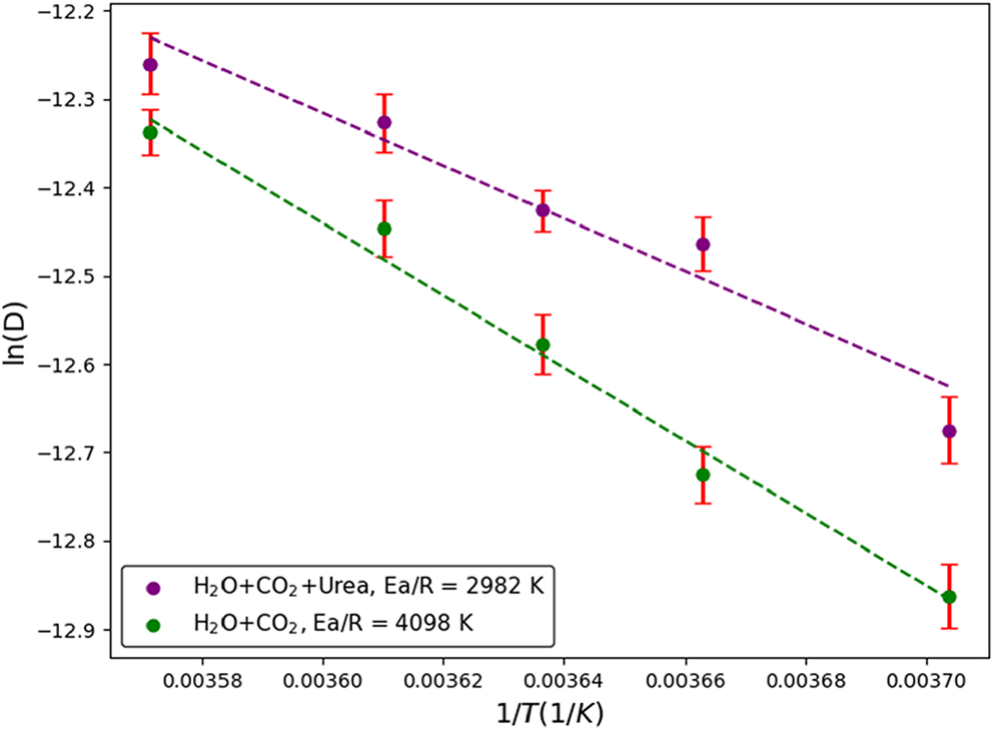
Variation of the natural logarithm of the self-diffusivity of CO_2_ as a function of the reciprocal temperature in different systems: water with dissolved CO_2_ (green circles), and water with dissolved CO_2_ and urea (purple circles).

**Table 3:**
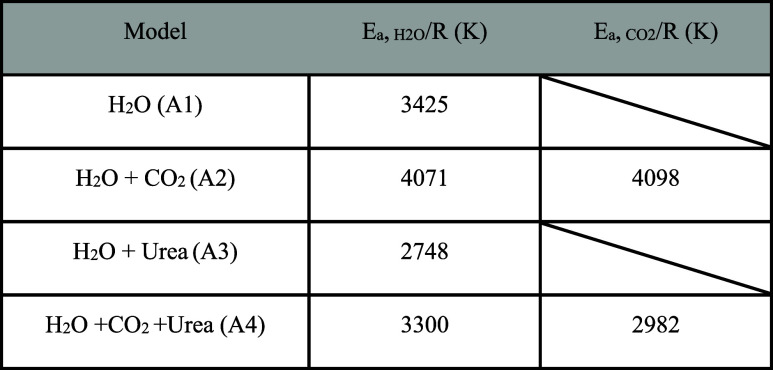
Diffusion Energy Barrier of H_2_O and CO_2_ Molecules

### 3.3 Urea Disrupts Nearby Water–Water H-Bonds Structure

In the previous section, we have observed that the enhanced molecular diffusion of water and CO_2_ near urea are consistent with the lowered diffusion barriers when urea is present. In this section we analyze the changes in water-dimer structures using Models A1–A3 and C1. All simulations were performed at 280 K and 45 bar for 50 ns. For each saved snapshot, all water pairs that meet the typical hydrogen-bond criteria are collected for further structural analysis. Here, a hydrogen bond is identified by an O–H···O angle θ < 30° and an O···O distance *r* < 0.35 nm (Note that a typical hydrogen bond is identified if *r* < 0.36 and θ < 30 at ambient conditions).^43^ However, since such geometric criteria of hydrogen bonding do not reflect its population across different *r* and θ,^44^ we also employed the elliptical boundary proposed by Wernet et al.,^45^ and evaluated hydrogen-bond propensities from our MD calculations. In this approach, the hydrogen-bond propensity, the ratio of the observed probability density to the probability density expected for a uniform spatial distribution, is determined as a function of *r* and θ. A propensity value greater than unity indicates that hydrogen bonds are more likely to occur than expected from a uniform distribution.

Figure 4 shows the propensity distributions for H_2_O···H_2_O dimers at different O–H···O angles (θ) and O···O distances (*r*). In all systems, the distributions are centered around the first O···O RDF peak (∼0.276 nm) and small bond angles (θ < 10°), representing the high-propensity region for hydrogen bonding. This region is noticeably more populated in the perfect hydrate system (Figure 4a) than in the fluid aqueous systems (Figure 4b–d), indicating a more ordered hydrogen-bond network in the hydrate crystal compared to the aqueous solutions. To better illustrate how additives (CO_2_ and urea) affect the hydrogen-bond network between water molecules, we calculated the differences in the distributions relative to that of pure water Model A1 (Figure 4b), as shown in Figure 5. The results indicate that the addition of CO_2_ enhances the population of stronger water pairs, with higher propensity for small bond angles (θ ≈ 0°–10°) and O···O distances near the first RDF peak (*r* ≈ 0.27–0.29 nm). In contrast, urea disrupts the water–water hydrogen-bond network, resulting in reduced propensity in the same high-propensity region.

**Figure 4:**
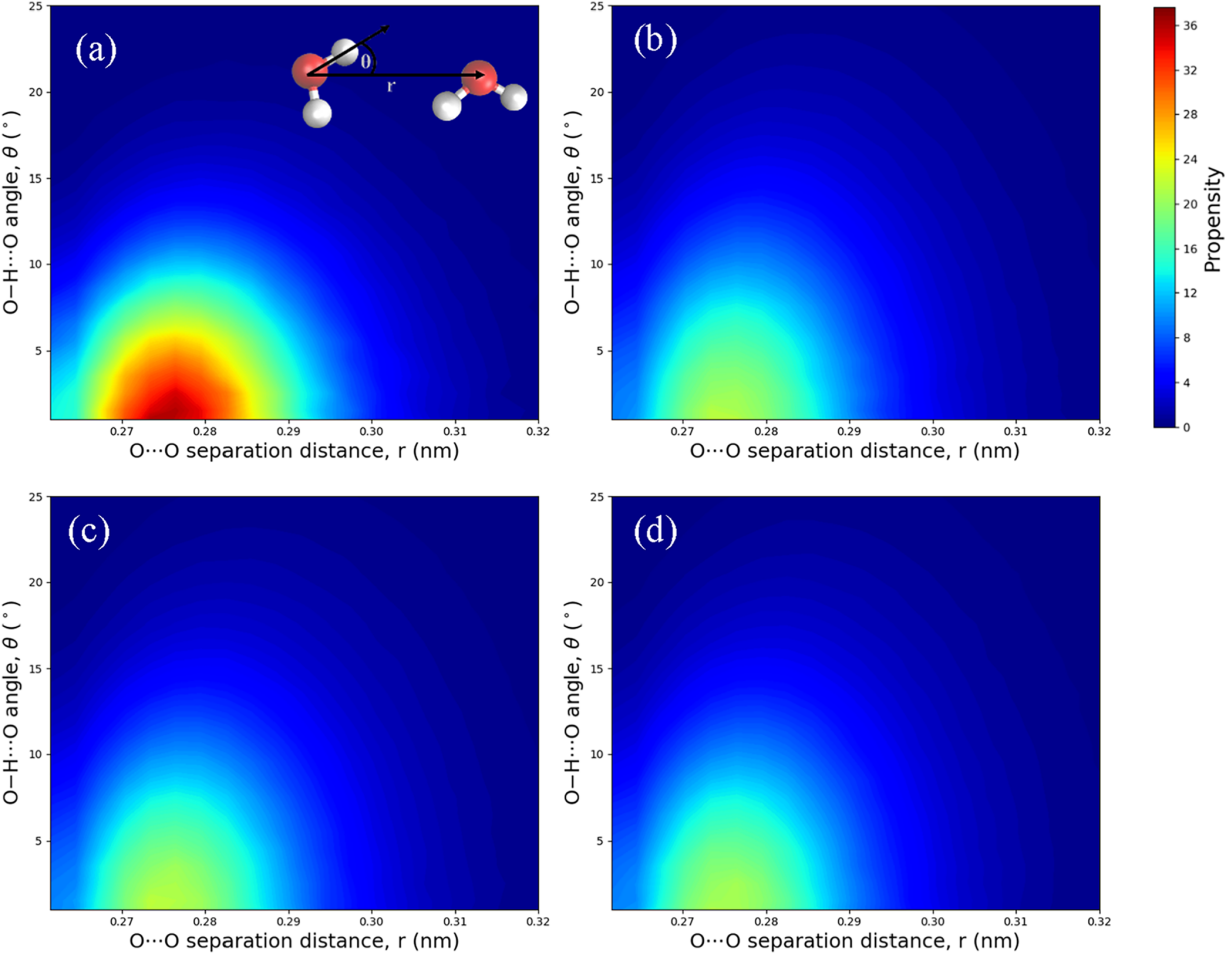
Propensity distributions of O–H···O angle (θ), and O···O distance (*r*) for H_2_O···H_2_O dimers: (a) perfect hydrate (model C1), (b) pure water system (model A1). (c) aqueous CO_2_ solution (Model A2), (d) aqueous urea solution (Model A3). In all cases, the propensity is highest at small bond (θ < 10°), with the O···O distances centered around the first RDF peak, approximately 0.276 nm.

**Figure 5:**
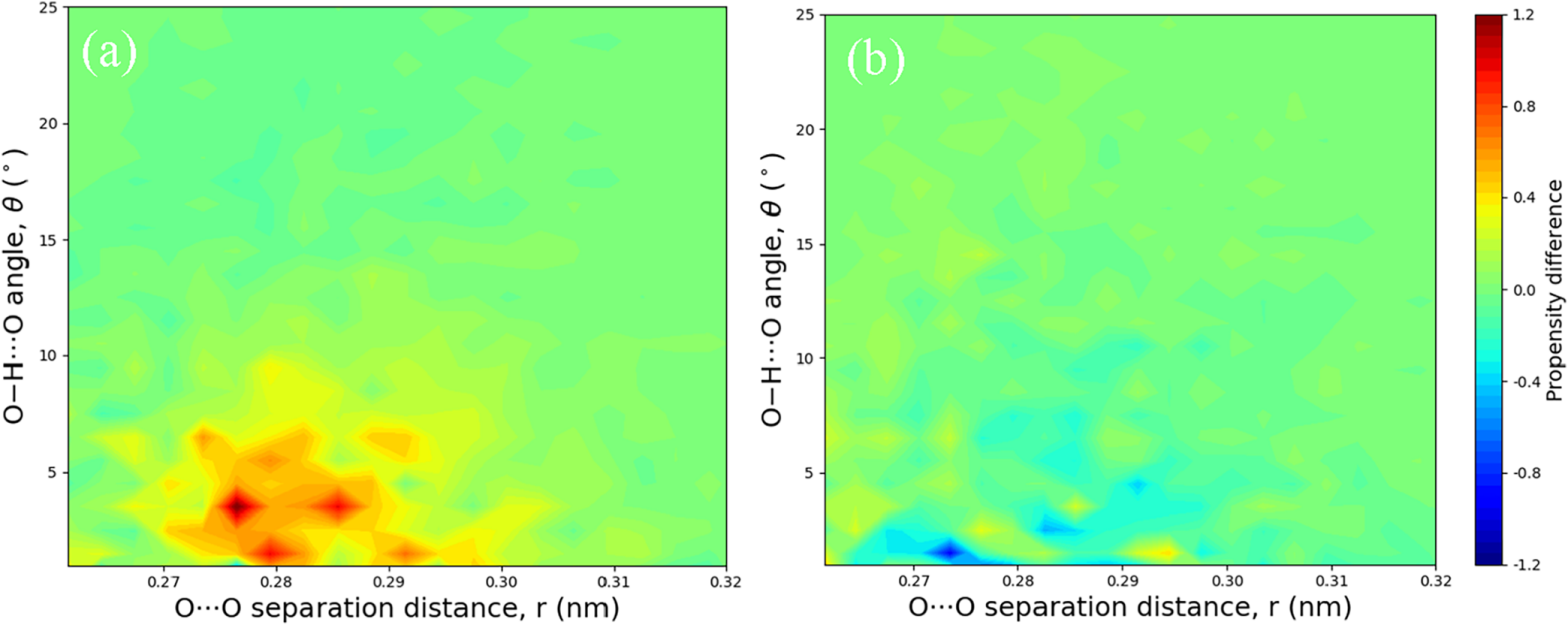
Propensity differences in O–H···O angle (*θ)*, and O···O distance (*r)* distributions for H_2_O···H_2_O dimers in different aqueous systems, calculated relative to the pure water system (Model A1): (a) aqueous CO_2_ solution (Model A2–A1), where the addition of CO_2_ increases the population in the high propensity region (*r* ≈ 0.27–0.29 nm, θ < 10°), and (b) aqueous urea solution (Model A3–A1), where the addition of urea reduces the population in the high propensity region.

A similar analysis was performed for the hydrogen-bond network between H_2_O and urea. As shown in Figure 6a, the propensity distribution of urea–water hydrogen bonds is generally more dispersed compared to that of pure water. This trend is more clearly illustrated in the contrast distribution in Figure 6b, calculated relative to the pure water system (Model A1), where almost the entire high-propensity region observed in the aqueous system is reduced. The results indicate that urea–water pairs exhibit larger D­(hydrogen donor)···A­(hydrogen acceptor) distances and more nonlinear bond angles compared to H_2_O–H_2_O pairs, which contributes to the overall more dispersed propensity distribution. This greater dispersion arises from the more diverse hydrogen-bond configurations between urea and water, as further illustrated in Figure 7 (see also the radial distribution function analysis for urea–water interactions in Figure S13 of the Supporting Information).

**Figure 6:**
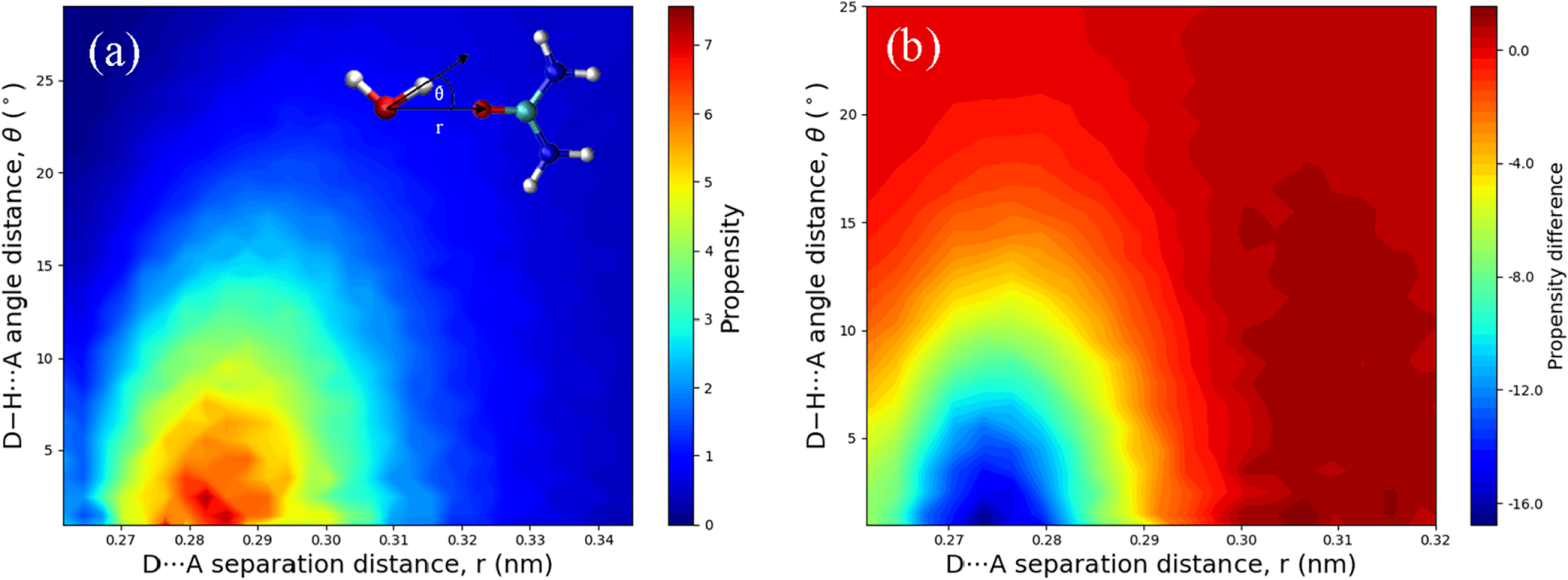
(a) Propensity distribution of the hydrogen-donor (D)–H···acceptor (A) angle (θ) and D···A distance (*r*) for urea···H_2_O dimers in aqueous urea solution (Model A3). The propensity is highest at small bond angles (θ < 15°) and D···A distances around 0.27–0.31 nm. (b) Differences in propensity distributions of D–H···A angle (θ) and D··*·*A distance (*r*) for urea···H_2_O dimers in aqueous urea solution relative to the pure water system (Model A3–A1). The high-propensity region shifts to larger bond angles and longer distances compared with the pure water system.

**Figure 7:**
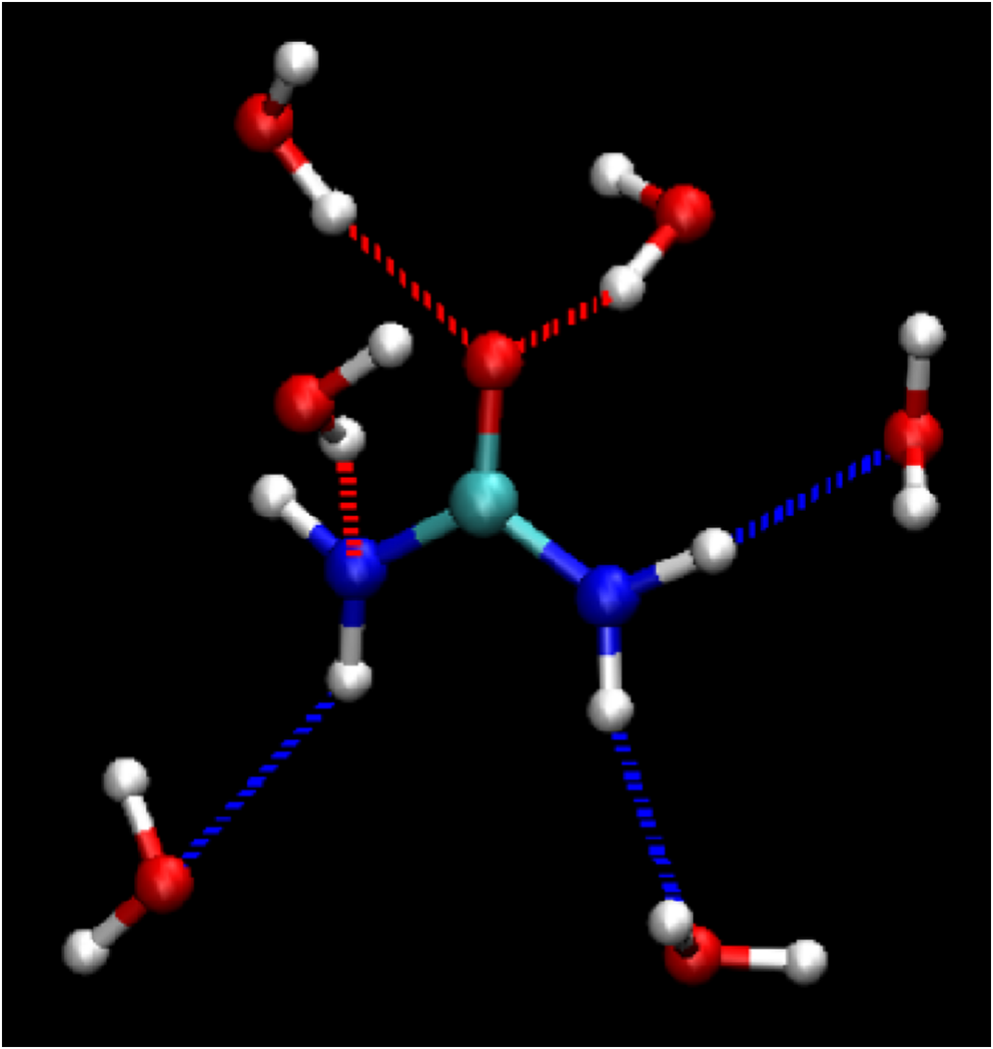
Schematic diagram of the hydrogen-bonding network involving urea and H_2_O.

### 3.4 Urea Weakens the Binding Strength between Nearby H_2_O

Here we further investigate the binding strength between water–water and water–urea pairs. Similar to the previous section, molecular pairs (water–water dimer and water-urea dimer) are identified from 50 ns NPT simulations of Models A1, B1, B2, and C1. For each simulation snapshot, all molecular pairs separated by less than 0.4 nm were identified and subjected to binding energy calculations. These calculations involved single-point energy evaluations of the molecular pair and each individual molecule in vacuum.^46^ The distribution of binding energy between water–water pairs in different system is shown in Figure 8. As can be seen water–water binding energy exhibit two peaks, a strong binding (−20 to −30 kJ/mol) and a weak binding (−2 to −10 kJ/mol). The strong binding is mostly electrostatic in nature, and the water pairs often meet the typical geometric criteria for hydrogen bond (*r* < 0.35 nm and θ < 30°); whereas the weak binding is due to van der Waals interactions and the water pairs do not meet both the distance and angle criteria. Figure 9 compares the distribution of binding energy between water–water and water-urea pairs in the urea aqueous solution system. As can be seen that the distribution of water–urea interactions are more broadly distributed and centered around higher values (approximately −10 to −20 kJ/mol), suggesting that these interactions are weaker than water–water interactions.

**Figure 8:**
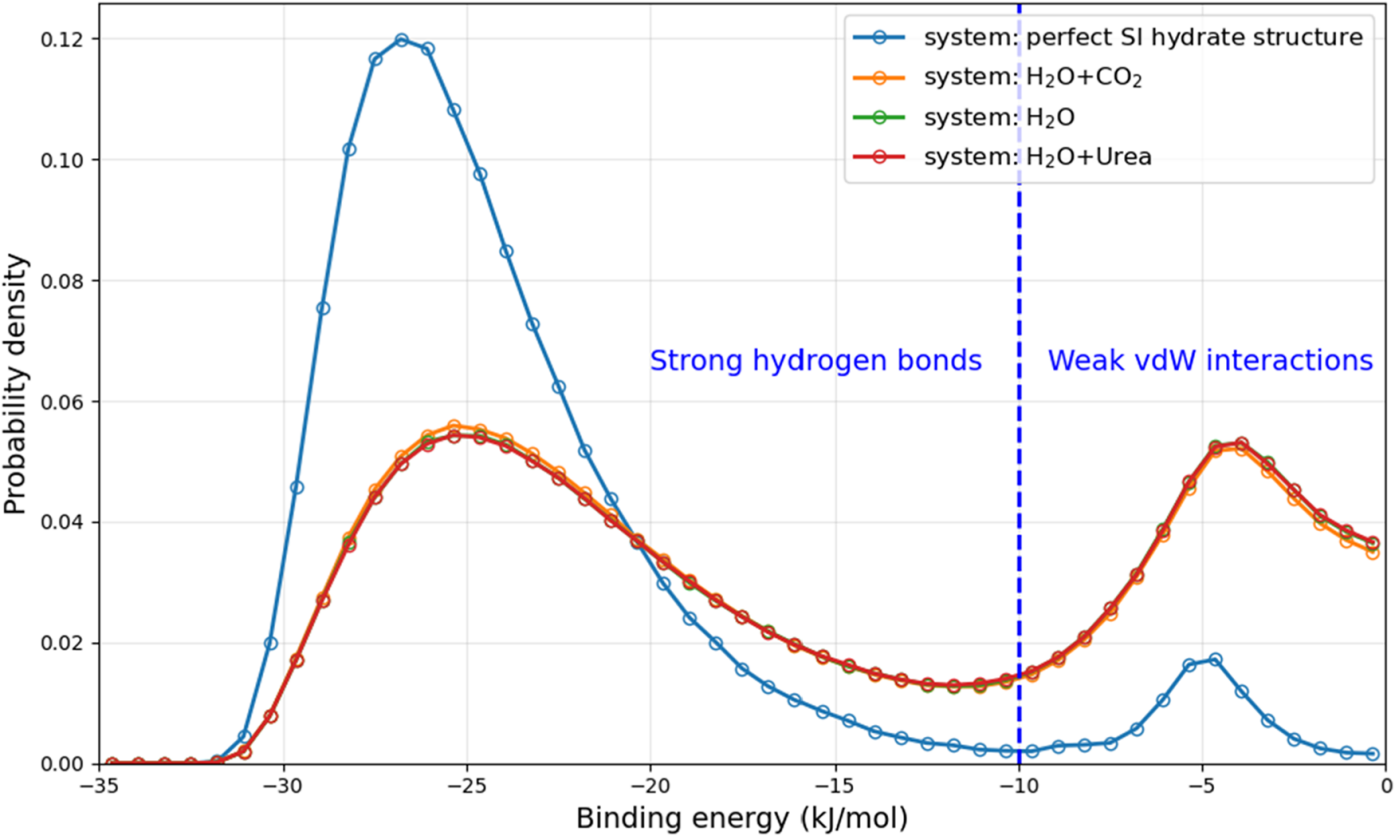
Distribution of binding energy for water–water molecular pairs in different systems: blue line—perfect S1 hydrate; orange line—H_2_O–CO_2_ system; green line—pure H_2_O system; red line—H_2_O–urea system.

**Figure 9:**
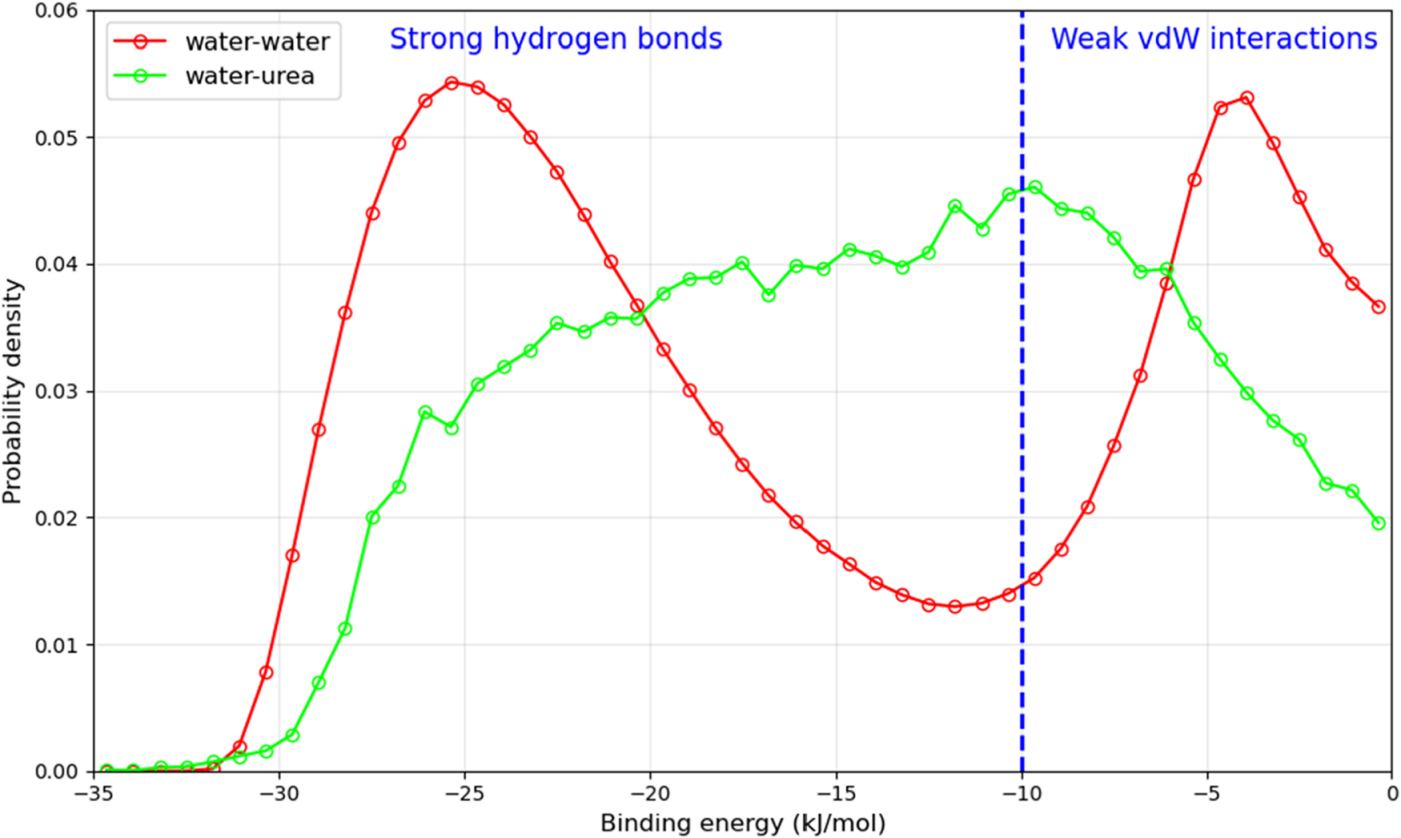
Distribution of binding energy for water–water and water-urea pairs for H_2_O + urea system (model B2).

To calculate the average hydrogen-bond energy, we adopt the hybrid distance–energy criterion^43^ for hydrogen-bond identification. Candidate hydrogen-bonded pairs are first selected using a distance cutoff of 0.4 nm, without applying any angular constraints. These pairs are then filtered based on an energy threshold of −10 kJ/mol: only those with binding energies stronger than this threshold are retained. Dimers with binding energies weaker than −10 kJ/mol are excluded from the average hydrogen-bond energy calculation, as such interactions often fail to simultaneously satisfy the typical geometric criteria for hydrogen bond. In Model C1, the perfect hydrate system, the average water–water hydrogen-bonding energy is approximately −24.67 kJ/mol. In Model A1­(pure water), this value is −22.09 kJ/mol. In Model A2 (H_2_O + CO_2_), the average energy slightly increases in magnitude to −22.15 kJ/mol. In Model A3 (H_2_O + urea), where urea competes with water for hydrogen bond, the average water–water hydrogen-bonding energy slightly decreases to −22.08 kJ/mol, while the average water–urea hydrogen-bonding energy is −18.39 kJ/mol.

To further assess the local influence of urea on water, we applied the spatial-decomposition method to compute hydrogen-bonding energies. The results in Figure 10a show that in the first solvation shell of urea (<0.40 nm), the average water–water hydrogen-bonding energy decreases to −21.88 kJ/mol, compared to −22.09 kJ/mol in the bulk water region. This suggests that urea weakens the strength of nearby water–water hydrogen bonds. On the other hand, Figure 10b indicates the presence of CO_2_ does not significantly alter the number of hydrogen bonds among water molecules but enhances their hydrogen-bonding energy to −22.41 kJ/mol, compared to −22.10 kJ/mol in the bulk water region due to the formation of hydrate-like structures.

**Figure 10:**
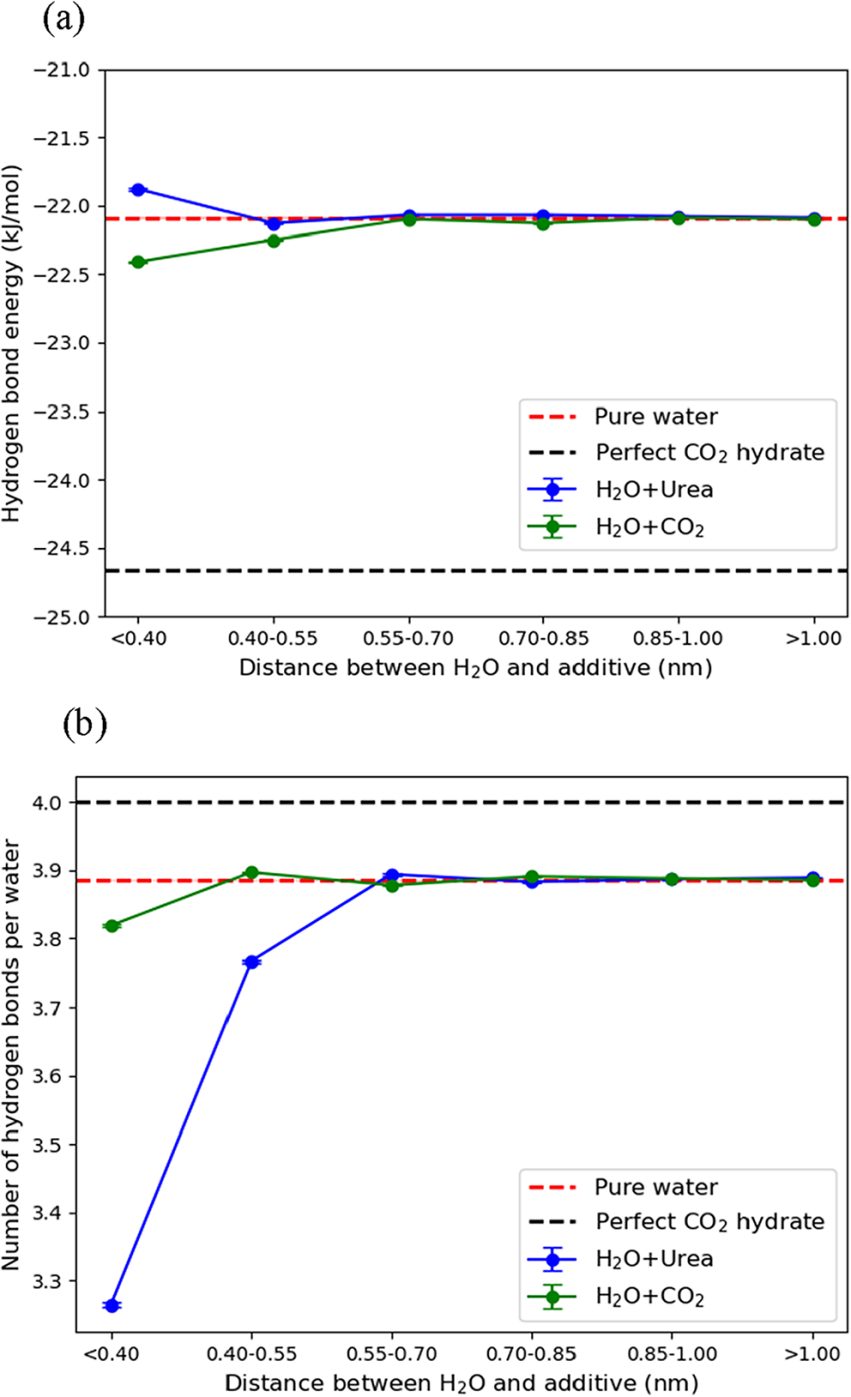
Averaged hydrogen-bond energy (a) and number of hydrogen bonds per water (b) as a function of distance from urea (blue circles) or CO_2_ (green circles). The corresponding values for pure water (red dashed horizontal line) and perfect CO_2_ hydrate (black dashed horizontal line) are also shown for comparison.

### 3.5 Urea Promotes Hydrate-like Structure Formation

The four-body (F_4_) order parameter has been widely used to distinguish water molecules in different phases or structural environments.^47^ It is defined as

4 $$F_{4}=\cos\left(3\Phi \right)$$

where ϕ is the H–O···O–H dihedral angle formed by the oxygen atoms and the outermost hydrogen atoms of a water dimer separated by less than 3.5 Å. In pure liquid water, the average *F*_4_ value is approximately zero, while water molecules in structure I (sI) hydrate exhibit *F*_4_ values ranging from 0.6 to 1.0, as shown in Figures S17 and S18 of the Supporting Information.

The distributions of *F*_4_ values for water molecules in Models A1–A4 are shown in Figure 11. As expected for homogeneous liquid systems, all distributions exhibit a bell-shaped curve centered around zero. However, the presence of CO_2_ (green circles) increases the population of water molecules with *F*_4_ values in the range of 0.6–1.0 (see Figure 11b), compared to pure water (blue circles). This shift indicates an increase in hydrate-like structuring, consistent with CO_2_ acting as a guest molecule in hydrate formation. Interestingly, the addition of urea (yellow circles) also leads to an enhanced population in the same *F*_4_ range, despite urea’s known role as a thermodynamic inhibitor of CO_2_ hydrates. This counterintuitive observation may be attributed to the weaker but multiple hydrogen bonding sites of urea, which result in a slightly higher fraction of local water arrangement similar to that of clathrate hydrate. Note that the *F*_4_ parameter is not directly dependent on hydrogen-bond geometry; therefore, hydrate-like ordering can persist even if the hydrogen-bond strength is locally weakened near urea molecules.

**Figure 11:**
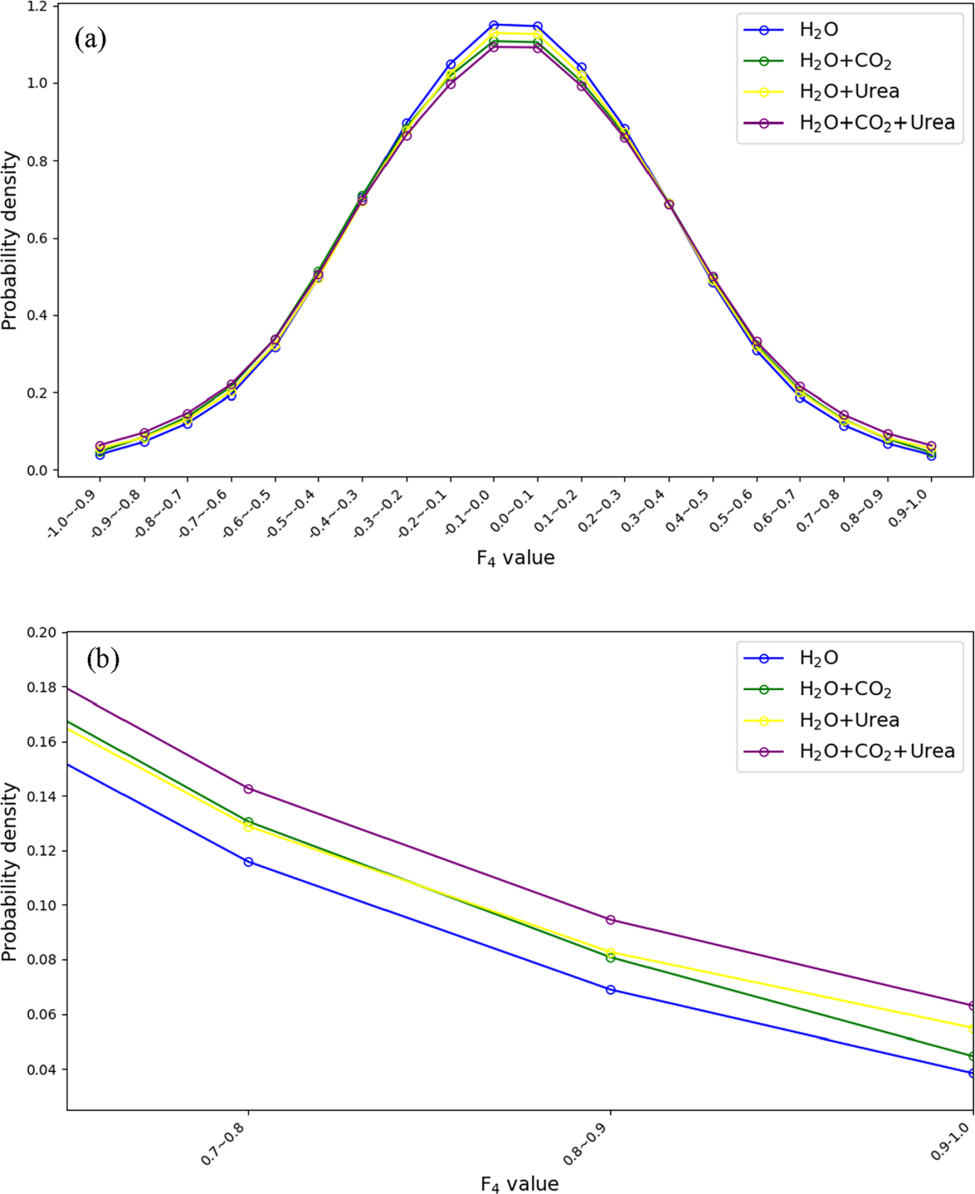
Distribution of *F*_4_ values for different homogeneous systems: (a) full range, (b) zoomed-in view.

It is important to note that CO_2_ hydrate crystallization typically proceeds through a nucleation step followed by crystal growth. Both the thermodynamic driving force (i.e., the chemical potential difference between the hydrate phase and the metastable solution) and the free-energy penalty associated with forming a solid–fluid interface influence the kinetics of nucleation and growth, in addition to mass transport. While our results demonstrate that urea enhances the diffusivity of CO_2_ and water, a comprehensive understanding of its role as a kinetic promoter also requires examining its effect on the nucleation free-energy landscape. Such an investigation, however, lies beyond the scope of the present work and will be more appropriately addressed in a future study.

## 4 Conclusions

In this study, molecular dynamics simulations were employed to investigate the effects of urea on water structure, energetics, and dynamics under CO_2_ hydrate formation conditions (270–280 K and 45 bar). The results reveal that urea locally enhances water diffusivity, particularly within its first solvation shell. Spatial decomposition analysis shows that this enhanced mobility is confined to the urea-affected region, with water dynamics returning to bulk-like behavior beyond approximately 1.00 nm. These findings are consistent with the observed reduction in diffusion energy barriers for both water and CO_2_ in urea-containing solutions.

Hydrogen-bond analysis indicates that urea competes with water for hydrogen-bonding partners, leading to a slight disruption of the local water–water hydrogen-bond network. The urea–water hydrogen bonds are weaker and more broadly distributed—with respect to both donor–acceptor separation and angular alignment—compared to those between water molecules. Structural characterization using the four-body (*F*_4_) order parameter reveals an increased population of hydrate-like water motifs in the presence of urea. This seemingly paradoxical behavior—coexisting weakened hydrogen bond and enhanced hydrate-like structuring—may arise from the hydrogen-bonding interactions of urea, which promote local cage-like water arrangements.

Overall, the combination of weakened water–water hydrogen bond, enhanced water mobility, and increased local hydrate-like structural ordering supports the role of urea as a kinetic promoter of CO_2_ hydrate formation. This molecular-level understanding provides new mechanistic insights into how urea molecules modulate water structure and dynamics to facilitate gas hydrate formation.

## Supplementary Material



## Notes

Simulation details; simulation model; force field verification; radial distribution function; *F*_4_ order parameter in structure I hydrate (PDF)
