# Supplementary material for: The Surprising Role of Urea in Promoting CO2 Hydrate Formation: Enhanced Molecular Diffusivity via Weakening of the Hydrogen-Bond Network
Source: J Phys Chem B. 2025 Sep 26;129(40):10440–50. doi: 10.1021/acs.jpcb.5c04817 (PMC12516713; doi:10.1021/acs.jpcb.5c04817)
Supplement: Supplementary file 1 [file jp5c04817_si_001.pdf]

Supporting Information for

The Surprising Role of Urea in Promoting CO_2 Hydrate Formation: Enhanced Molecular Diffusivity via Weakening of the Hydrogen-Bond Network

Jun-Wei Hsu[1], David T. Wu[1,2] and Shiang-Tai Lin[1*]

[1]Department of Chemical Engineering, National Taiwan University, Taipei 106319, Taiwan

[2]Institute of Chemistry, Academia Sinica, Nangang, Taipei 115201, Taiwan

1. Simulation Details

The non-bond terms include van der Waals interaction and coulomb interaction. The van der Waals interaction describes the exchange-repulsive and dispersive interactions. We used the Lennard-Jones 12-6 function to describe the van der Waals interaction in our simulation. The equation is described in Eq 3.5.

Normally, the geometric combination rules for the Lennard - Jones parameters between different atoms are shown as Eq 3.6. However,the interaction between water and CO_2 is too weak compared to the real world. We introduce another parameter called scaling factor to adjust van der Waals force Eq 3.7. In this work we set both $X_{water\text{-}CO2}$ and $X_{water\text{-}urea}$ to 1.1. [1]

$$\epsilon_{ij} = \sqrt{\epsilon_{ii}\epsilon_{jj}} \tag{3.6}$$

$$\epsilon_{ij} = X\sqrt{\epsilon_{ii}\epsilon_{jj}} \tag{3.7}$$

Table S1. Force field parameters.

Molecule	Atom	ε (kJ/mol)	σ (nm)	q (e)
**water**	O	0.881949	0.316685	0
	H	0	0	0.5897
	MW	0	0	-1.1794
**CO_2**	C	0.233865	0.2757	0.6512
	O	0.669335	0.3033	-0.3256
**urea**	C	0.43932	0.375	0.142
	O	0.87864	0.296	-0.390
	N	0.71128	0.325	-0.542
	H	0	0	0.333

2. Simulation Model

Different molecular models, as summarized in Table S1 and in Figs. S1–S3, were created with Materials Studio[2]. for study different aspects of the urea-CO_2-water ternary mixture.

Table S1 Molecular models used in this

Model	System	Condition	Studied Properties
A1	1500 H_2O	270,273,275, 277,280 K and 45 bar	Self-diffusivity, Hydrogen bonds, Hydrate-like structure
A2	1500 H_2O + 60 CO_2		
A3	1500 H_2O + 63 urea (w_{urea}= 12.3 wt%)		
A4	1500 H_2O + 63 urea + 60 CO_2 (w_{urea}= 12.3 wt%)		
B1	1000 H_2O + 10 urea (w_{urea}=3.2 wt%)	280 K and 45 bar	Distance-dependent water–additive interactions
B2	1000 H_2O + 10 CO_2		

C1	368 H_2O + 64 CO_2		Perfect hydrate

Note: Urea weight percent is defined as $w_{urea}= m_{urea}/(m_{urea}+m_{H2O}) \times 100$ %.

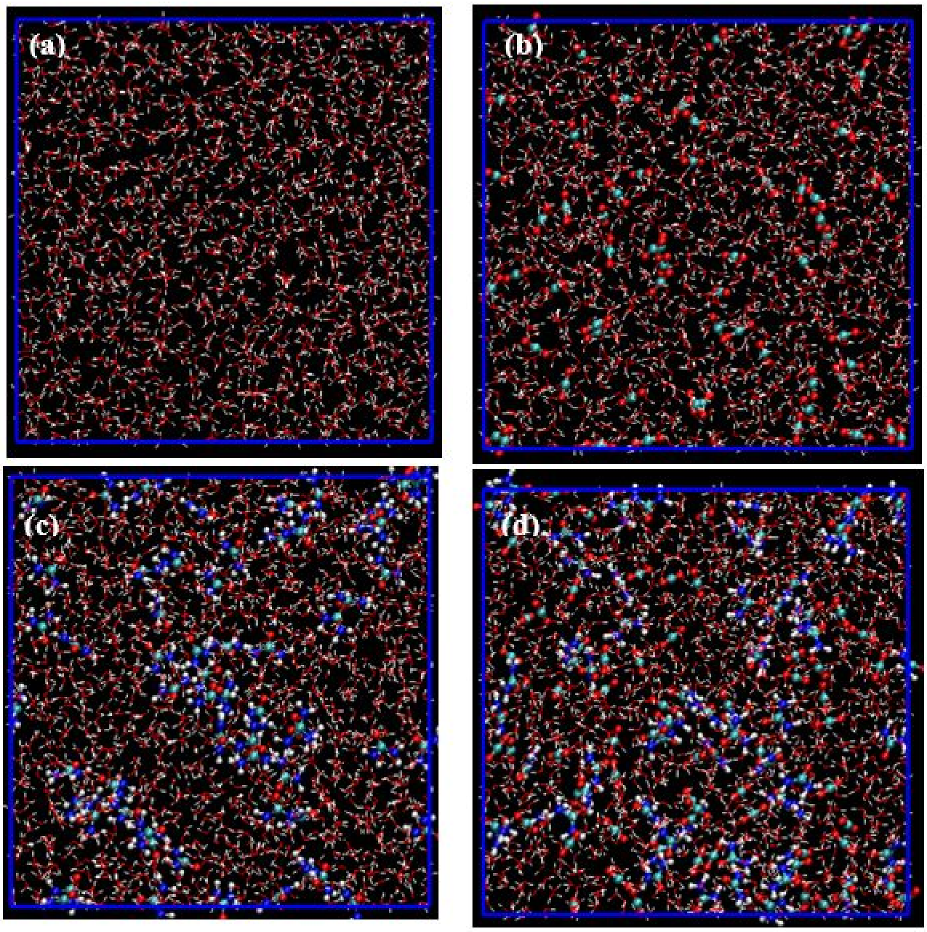

Fig. S1. Initial structures used in the simulations of Models A1–A4: (a) Model A1, (b) Model A2, (c) Model A3, (d) Model A4.

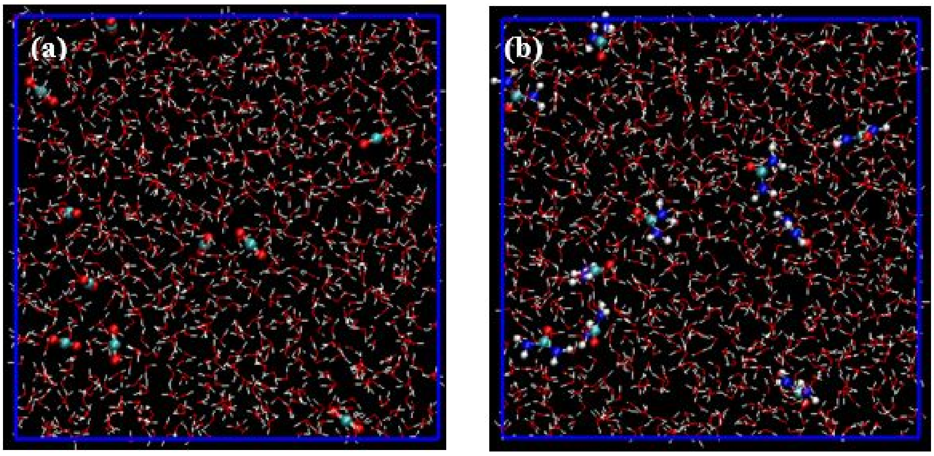

Fig. S2. Initial structures used in the simulations of Models B1–B2: (a) Model B1, (b) Model B2.

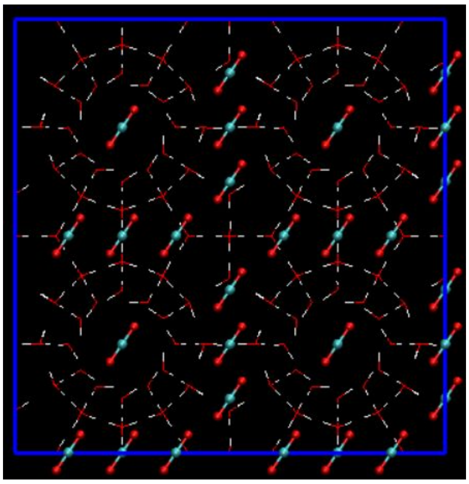

Fig. S3. The initial structures used for simulation of 2 × 2 × 2 supercell of perfect structure I CO_2 clathrate hydrate.

3 Force Field Verification

In this section, we validate the force fields used for H_2O and CO_2. The force field for urea has been previously validated in Refs.[1, 3]

3.1 Dissociation Heat

The dissociation heat is a key thermodynamic property of gas hydrates, and its accurate determination is essential for the reliability of force fields used in hydrate dissociation simulations. In this study, Hess's law was applied to calculate the dissociation heat at 282 K and 36 bar over a 50 ns simulation period. The system was divided into three models: a perfect S1 3×3×3 (Fig. S4 (a)), a liquid water phase saturated with CO_2 (Fig. S4 (b)), and the CO_2 gas phase (Fig. S4 (c)). The dissociation heat was determined as the difference in enthalpy between the final state and the initial hydrate phase, specifically expressed as ΔH = H (Fig. S4 (b)) + H (Fig. S4 (a)) – H (Fig. S4 (c)). Our simulation results yielded a dissociation heat of 50.02 kJ/mol, which is approximately 8.8% lower than the experimental value of 56.85 kJ/mol [4]. This deviation is within an acceptable range and demonstrates the capability of our model to capture the essential thermodynamic behavior of hydrate dissociation. The slight underestimation may be attributed to the limitations of the force field used in the simulation, as well as the finite size of the simulation box.

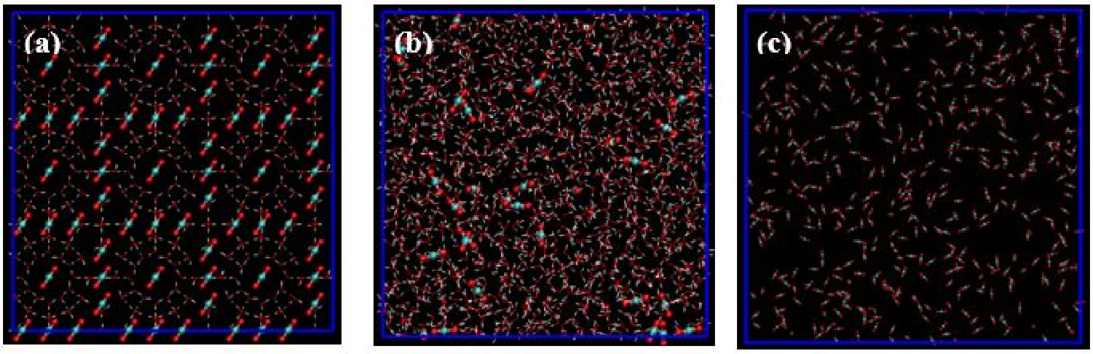

Fig. S4. The initial structures used for simulation of dissociation heat (a) 3×3×3 perfect SI hydrate, (b) CO_2 aqueous solution and (c) pure water.

3.2 Solubility

For CO_2 solubility measurement, the initial system is shown as Fig. S5 and we ran the simulation at 283K and 45 bar for 300ns. To determine the solubility of CO_2 in pure water system, we plot the mole fraction distribution with distance. The CO_2 solubility from the MD simulation is 0.03408 (Fig. S6), which is 25% higher than the experimental result (mole fraction = 0.027).[5]

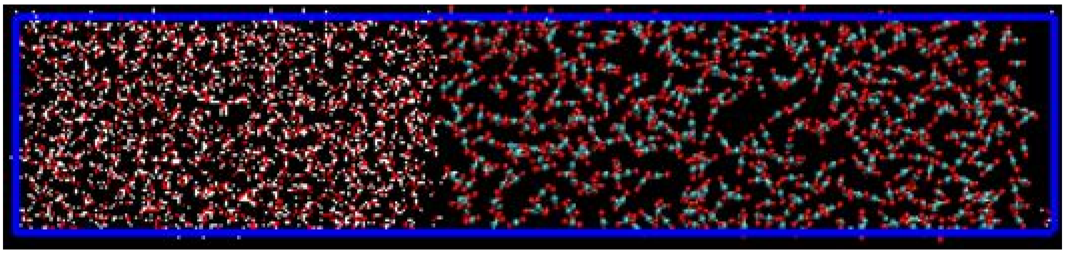

Fig. S5. The initial structures used for simulation of CO_2 solubility in water.

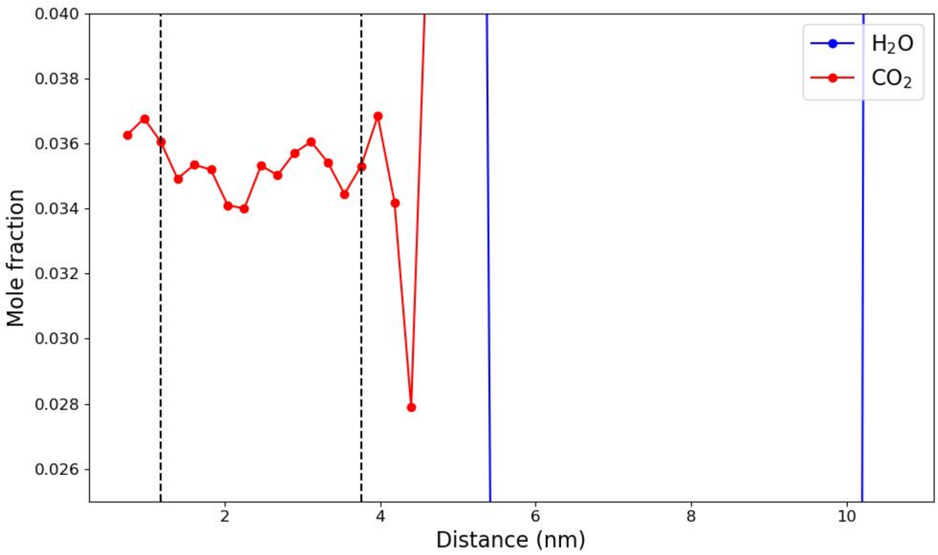

Fig. S6. The mole fraction distribution with distance from the NPT simulations of the two-phase model at 283 K and 45 bar.

3.3 Melting Point

As a matter of fact, potential energy will increase with time when hydrate starts melting, and potential energy will decrease when hydrate starts crystallize. Given this we can plot the evolution of the potential energy with time to find the melting point of hydrate. The initial structure of the system shows at Fig. S7. We performed four independent simulations, each lasting 300 ns, at pressures of 45 bar and 2500 bar across various temperature. The plot of potential energy change with time is shown as Fig. S6. The potential kept increasing for temperature higher than 286 K and the potential keep decreasing for temperature lower than 285 K. This means the melting point of CO_2 hydrate in the pure water system is between 285 K and 286 K (Fig. S8). The result is slightly higher than literature (T_{melt} =283 K). [6] We can observe the crystal growth and melting by comparing Fig. S9 and Fig. S10. Furthermore, we test whether the Tip4P-ice and EPM2 models can simulate the high-pressure conditions of hydrate nucleation and demonstrate the same simulation at 2500 bars for 300 ns, under the condition where the clathrate hydrate, water, and CO_2 coexist in three phases. The results are presented in Fig. S11, showing good agreement with the experimental values [7].

Table S1 Comparison of simulated and experimental melting points.

Property	This work	Exp
mp (K) at 45 bar	285 ± 1	283
mp (K) at 2500 bar	294 ± 1	293.5

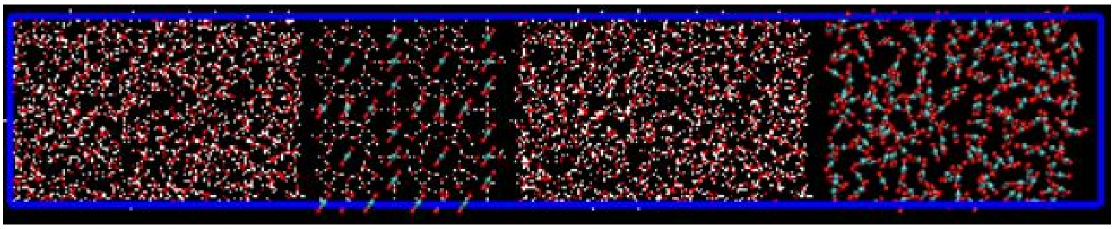

Fig. S7. The initial structure used for simulation of melting point (modelS4).

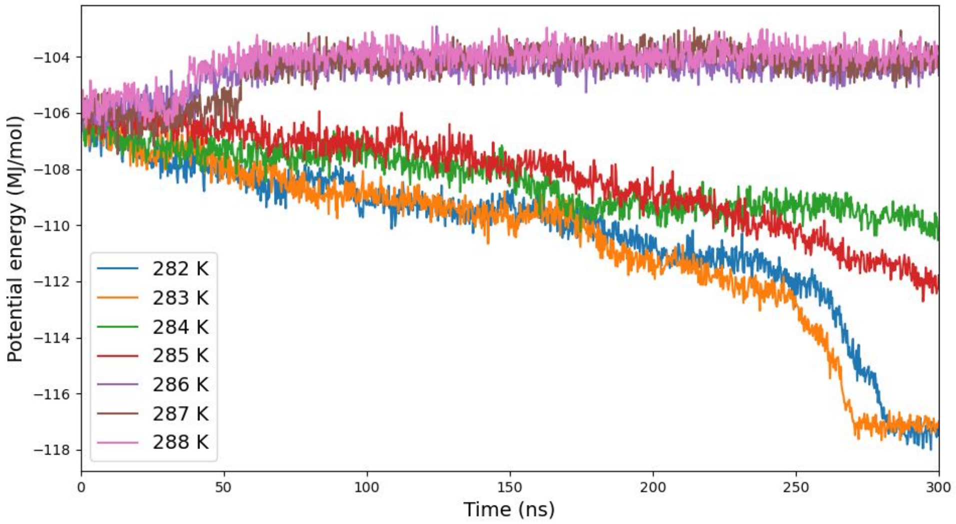

Fig. S8. The time evolution of potential energy from the NPT simulations of the three-phase CO_2 hydrate model at 45 bar and different temperatures.

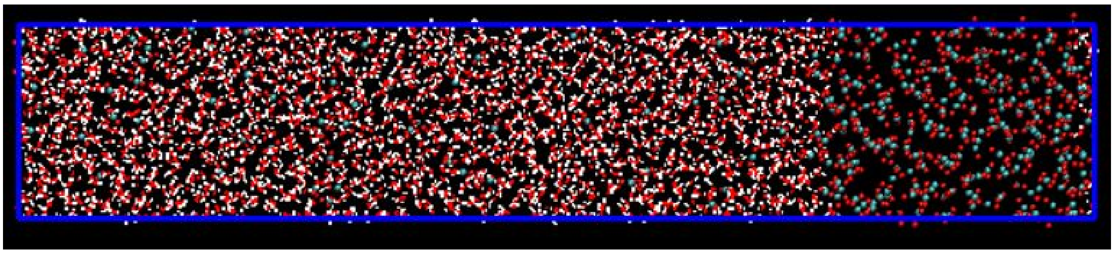

Fig. S9. The final equilibrium structure of the system at a temperature of 288 K and 45 bar.

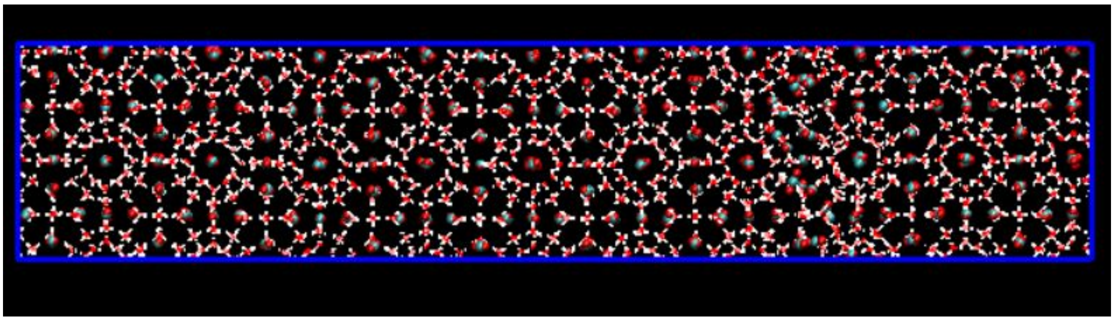

Fig. S10. The final equilibrium structure of the system at a temperature of 282 K and 45 bar.

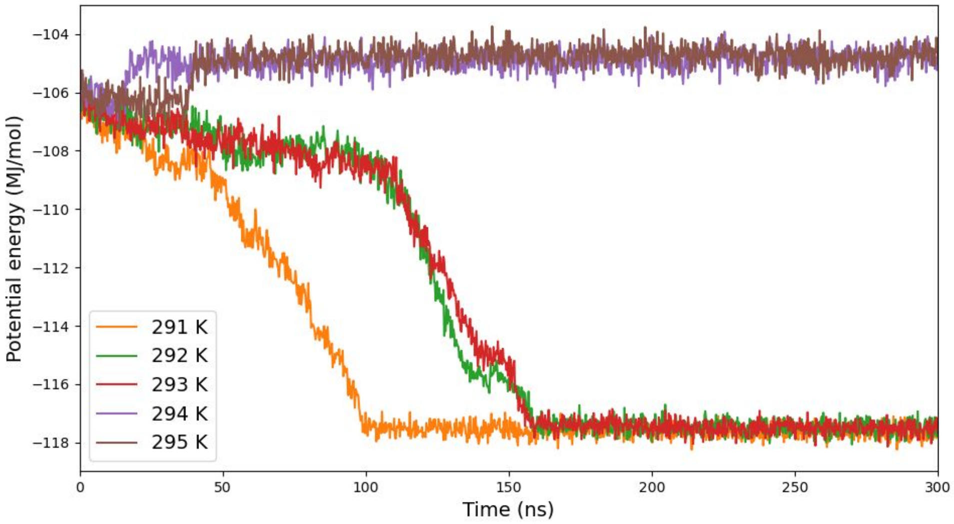

Fig. S11. The time evolution of potential energy from the NPT simulations of the three-phase CO_2 hydrate model at 2500 bar and different temperatures.

3.4 Diffusivity

Although the TIP4P/ICE water model has been reported to yield highly accurate predictions of ice and hydrate phase diagrams [8], it significantly underestimates dynamic properties such as the diffusion coefficient when compared to experimental values.[9] Nevertheless, in this study, we simulated water diffusivity under ambient conditions (298 K, 1 bar) to enable direct comparison with experimental measurements. We performed 10 independent simulations of 50 ns each, and computed the diffusivity from

the MSD averaged over these runs. The simulation yielded a water diffusivity of 1.169×10^{-9} m²/s in the absence of urea, and 1.1438×10^{-9} m²/s at a urea mole fraction of 0.12. While these values remain substantially lower than the experimental range (2.30 to 1.78×10^{-9} m²/s), the observed decreasing trend with increasing urea concentration is consistent with experimental observations [10].

4. Radial Distribution Function

In molecular simulations, the radial distribution function (RDF) is a powerful tool for characterizing the spatial range of intermolecular interactions, and is particularly important in the analysis of hydrogen bonding. In this section, we examine the intermolecular interactions among H_2O, urea, and CO_2 by analyzing RDFs from three systems: (1) a homogeneous water system (Model A1), (2) a homogeneous water–urea system (Model A2) at 280 K and 45 bar over 50 ns, and (3) a hydrate-containing system (Structure C1) at 282 K and 36 bar. These analyses provide insights into the nature and range of intermolecular force in hydrate-forming conditions.

Fig. S12 (Model A1) presents two primary RDF curves: the O(H_2O)–H(H_2O) pair (sky blue) and the O(H_2O)–O(H_2O) pair (blue). The first peaks appear at 0.185 nm and 0.276 nm, corresponding to the equilibrium hydrogen bond distances for O–H and O–O interactions, respectively. The O–O RDF (blue) decays gradually and reaches a plateau near 0.33 nm, indicating that the hydrogen bond cutoff distance should be no less than 0.33 nm. To accommodate thermal fluctuations and structural distortion, a cutoff of approximately 0.36 nm is typically applied in hydrogen bond analyses.

Fig. S13 (Model A2) presents three primary RDF curves: O(H_2O)–H(urea) (green), H(H_2O)–N(urea) (lime), and H(H_2O)–O(urea) (teal). Distinct peaks at 0.185 nm are observed in the green and teal curves, indicating that the carbonyl oxygen of urea acts as a hydrogen bond acceptor, while its nitrogen serves as a hydrogen bond donor. The

absence of a peak in the $H(H_2O)$–N(urea) curve (lime) suggests that the nitrogen atom does not function effectively as a hydrogen bond acceptor. This may be due to electrostatic repulsion from the two covalently bonded hydrogen atoms or an insufficient electronegativity difference between nitrogen and hydrogen to support strong hydrogen bonding.

If we examine the RDFs of $O(H_2O)$–O(urea) (red) and $O(H_2O)$–N(urea) (blue) (Fig. S14), we observe primary peaks at 0.282 nm and 0.35 nm, respectively, along with secondary peaks near 0.5 nm in both curves. Notably, the first peak of the blue curve is broader, reflecting a wider distribution of distances. This behavior arises from the intrinsic topology of the urea molecule. As illustrated in Fig. S15, when one of the urea atoms (O, N1, or N2) forms a hydrogen bond with a water oxygen atom, the $O(H_2O)$ atom is likely to be in close proximity to the other heteroatoms of the same urea molecule. The resulting geometric constraints of hydrogen bonding—both in terms of distance and angle—lead to a broader and more distributed RDF profile.

Finally, Fig. S16 (Model A3) presents two primary RDF curves for Structure 1 hydrate: the $O(H_2O)$–$H(H_2O)$ pair (sky blue) and the $O(H_2O)$–$O(H_2O)$ pair (blue). The position of the first peak is similar to that in Fig. S12, but significantly sharper, indicating more rigid hydrogen bonding. Additionally, the presence of periodic peaks in the range of 0.4–0.9 nm reflects the long-range order characteristic of a crystalline solid.

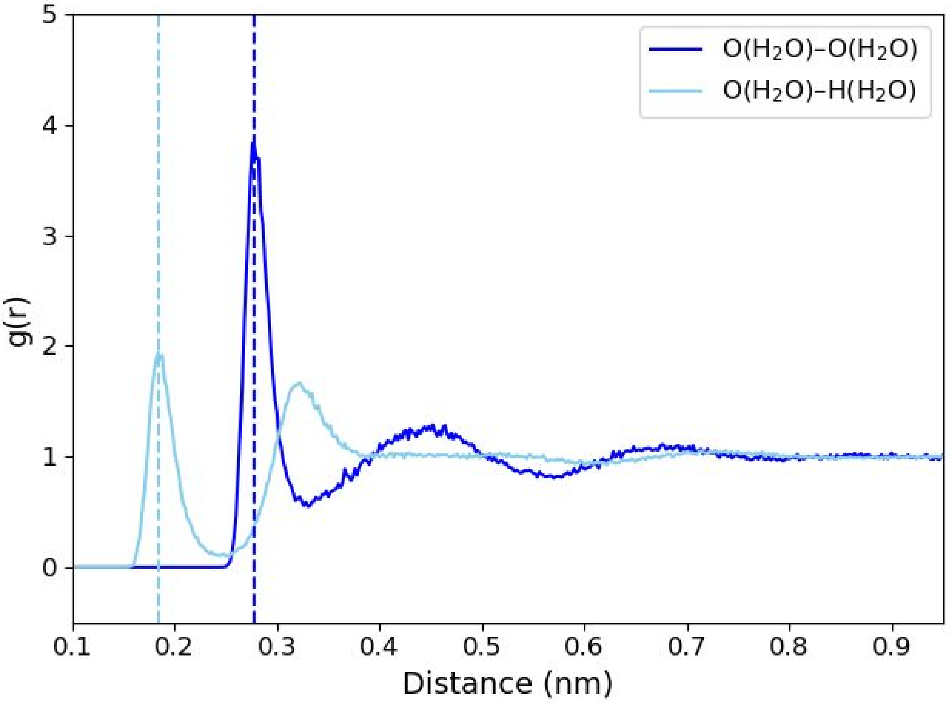

Fig. S12. Radial distribution function of pure water system.

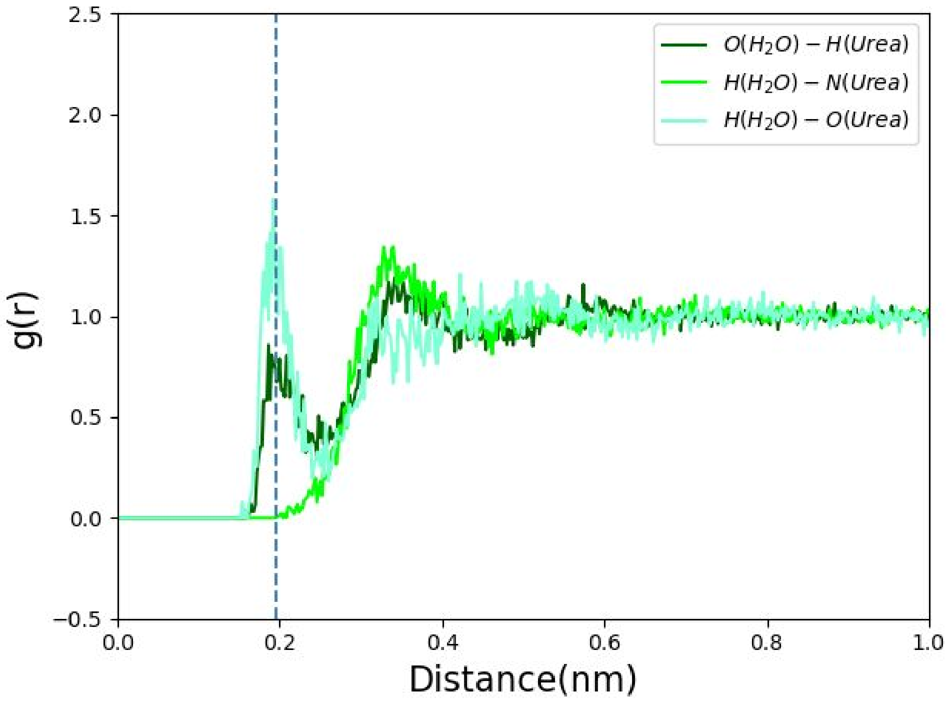

Fig. S13. Radial distribution function of water/urea system (acceptor-donor).

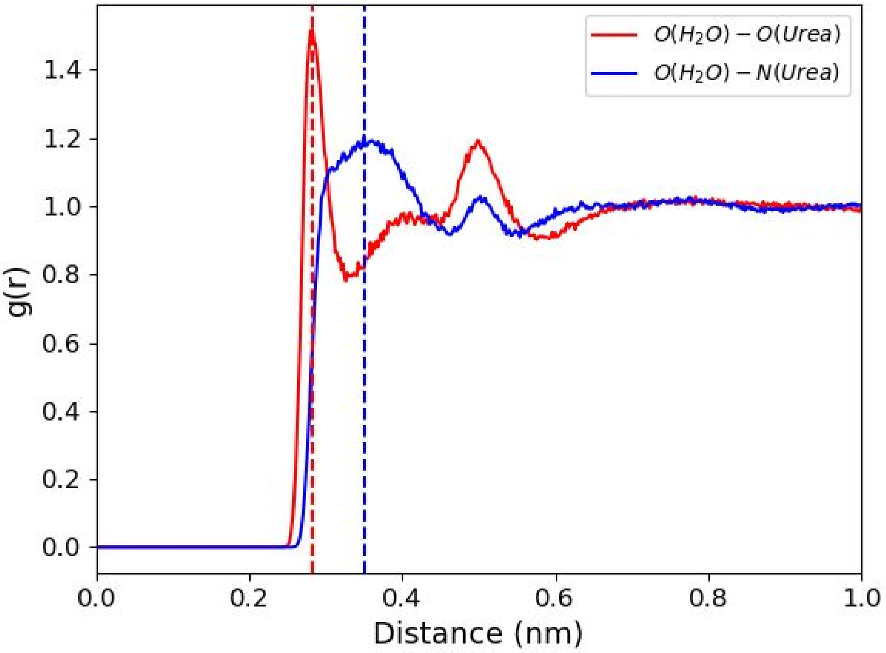

Fig. S14. Radial distribution function of water/urea system (acceptor-hydrogen).

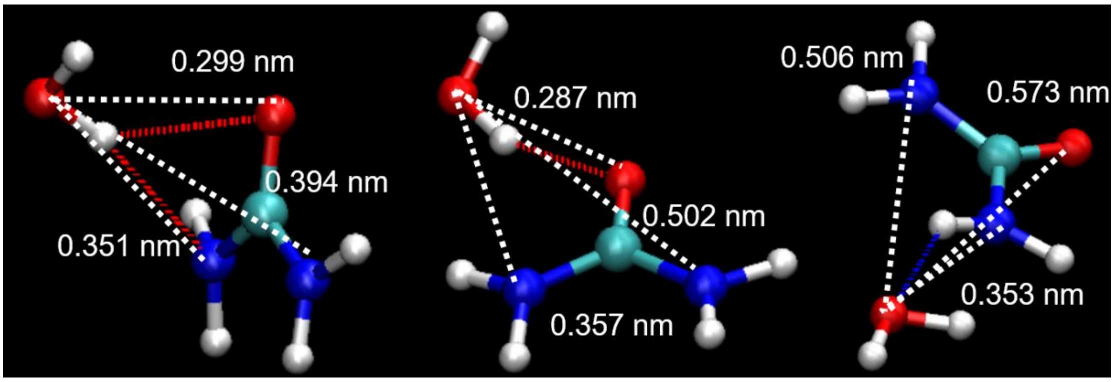

Fig. S15. Schematic representation of hydrogen bonding between water and urea. White lines indicate the separation distances between atoms, while red and blue lines denote hydrogen bonds formed between donor-H and acceptor atoms.

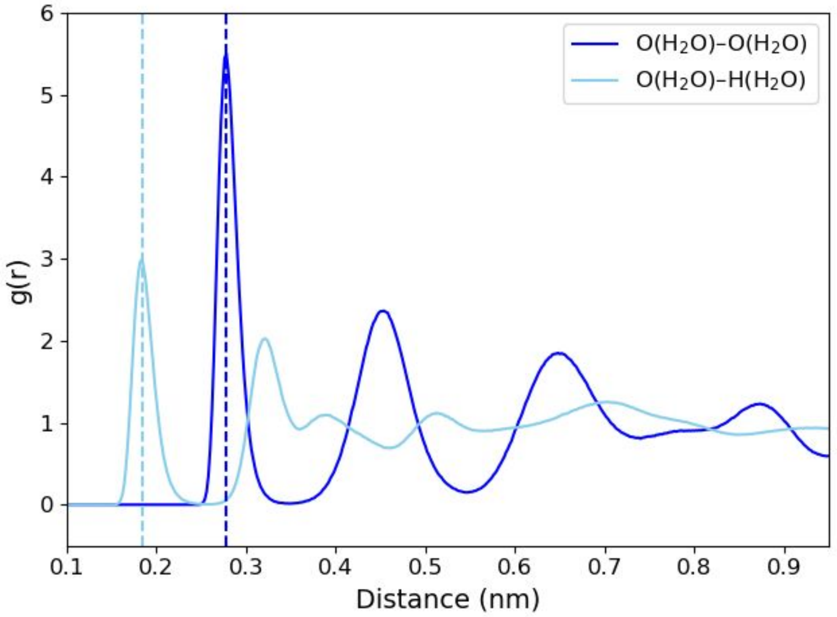

Fig. S16. The radial distribution function of perfect hydrate system (H_2O-H_2O).

5. F_4 Order parameter in Structure I Hydrate

Order parameters serve as a directed method to observe whether additives promote nucleation. In this section, we utilize the Four-Body Order Parameter (F_4) to investigate nucleation phenomena. The initial systems, denoted as Model A1 and C1, were simulated at 280 K and 45 bar for 50 ns. As shown in Fig. S17 and Fig. S18 , the average F_4 value is approximately 0 for pure liquid water and around 0.88 for a perfect structure I hydrate.

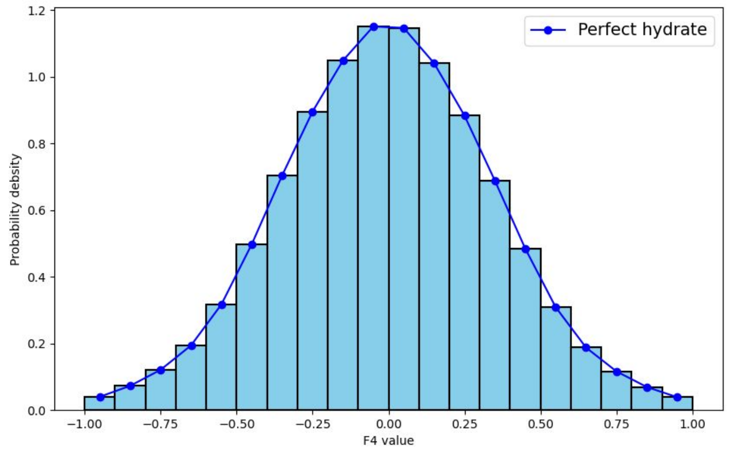

Fig. S17. The F_4 value distribution for liquid water.

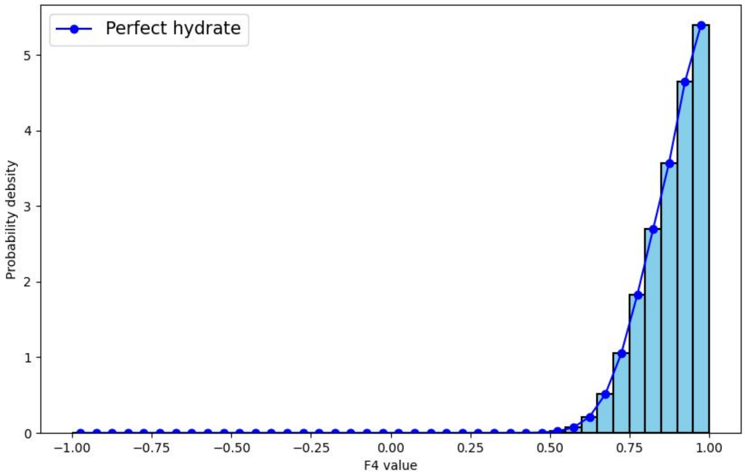

Fig. S18. The F_4 value distribution for perfect structure I hydrate.

Reference

(1) Wang, P.-W.; Wu, D. T.; Lin, S.-T. Promotion mechanism for the growth of CO 2 hydrate with urea using molecular dynamics simulations. *Chemical Communications* **2021**, *57* (43), 5330-5333.

(2) Module, F. Material Studio 6.0. *Accelrys Inc., San Diego, CA* **2011**.

(3) Duffy, E. M.; Severance, D. L.; Jorgensen, W. L. Urea: potential functions, log P, and free energy of hydration. *Israel journal of chemistry* **1993**, *33* (3), 323-330. Kokubo, H.; Pettitt, B. M. Preferential solvation in urea solutions at different concentrations: properties from simulation studies. *The journal of physical chemistry B* **2007**, *111* (19), 5233-5242.

(4) Sabil, K. M.; Witkamp, G.-J.; Peters, C. J. Estimations of enthalpies of dissociation of simple and mixed carbon dioxide hydrates from phase equilibrium data. *Fluid Phase Equilibria* **2010**, *290* (1-2), 109-114.

(5) Dodds, W.; Stutzman, L.; Sollami, B. Carbon dioxide solubility in water. *Industrial & Engineering Chemistry Chemical & Engineering Data Series* **1956**, *1* (1), 92-95.

(6) Adisasmito, S.; Frank III, R. J.; Sloan Jr, E. D. Hydrates of carbon dioxide and methane mixtures. *Journal of Chemical and Engineering Data* **1991**, *36* (1), 68-71.

(7) Nakano, S.; Moritoki, M.; Ohgaki, K. High-pressure phase equilibrium and Raman microprobe spectroscopic studies on the CO2 hydrate system. *Journal of chemical & engineering data* **1998**, *43* (5), 807-810.

(8) Abascal, J.; Sanz, E.; García Fernández, R.; Vega, C. A potential model for the study of ices and amorphous water: TIP4P/Ice. *The Journal of chemical physics* **2005**, *122* (23). Zhang, X.; Yang, H.; Huang, T.; Li, J.; Li, P.; Wu, Q.; Wang, Y.; Zhang, P. Research progress of molecular dynamics simulation on the formation-

decomposition mechanism and stability of CO2 hydrate in porous media: A review. *Renewable and Sustainable Energy Reviews* **2022**, *167*, 112820.
(9) Baran, Ł.; Rżysko, W.; MacDowell, L. G. Self-diffusion and shear viscosity for the TIP4P/Ice water model. *The Journal of Chemical Physics* **2023**, *158* (6). Picaud, S. Dynamics of TIP5P and TIP4P/ice potentials. *The Journal of chemical physics* **2006**, *125* (17).
(10) Mayele, M.; Holz, M. NMR studies on hydrophobic interactions in solution. Part 5. Effect of urea on the hydrophobic self-association of tert-butanol in water at different temperatures. *Physical Chemistry Chemical Physics* **2000**, *2* (10), 2429-2434.
